# Cardiac magnetic resonance mapping for the diagnosis of reverse ventricular Takotsubo cardiomyopathy

**DOI:** 10.1093/ehjci/jeac016

**Published:** 2022-02-01

**Authors:** Lorenzo Marcon, Andrea Baggiano, Gianluca Pontone

**Affiliations:** 1 Centro Cardiologico Monzino IRCCS, Via C. Parea 4, 20138 Milan, Italy; 2 Cardiovascular Section, Department of Clinical Sciences and Community Health, University of Milan, Milan, Italy

##  

**Figure:**
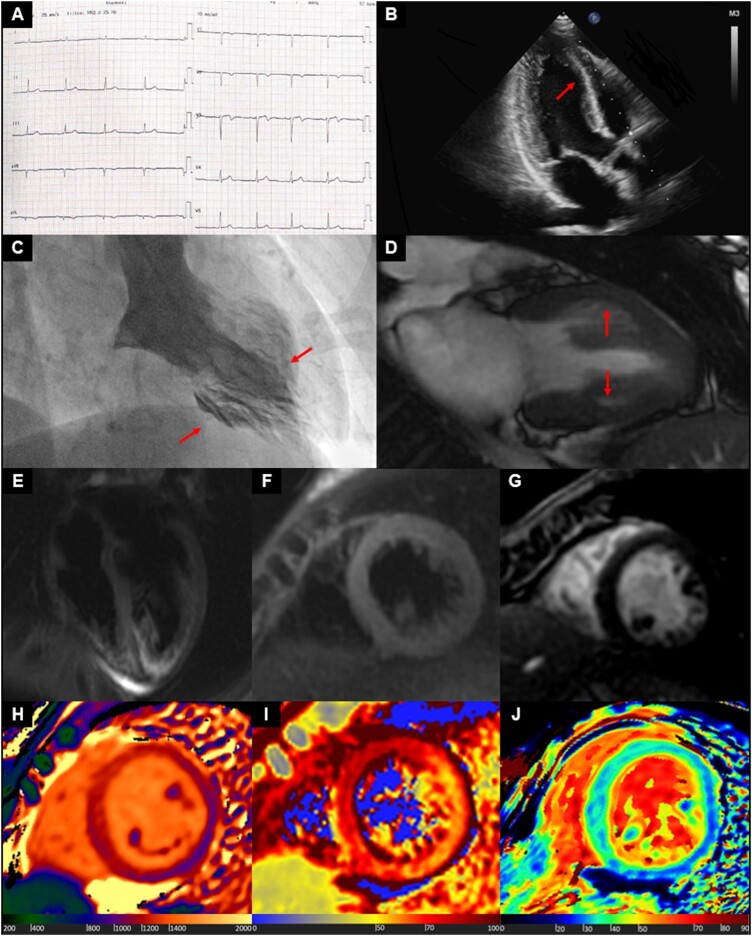


A 37-year-old woman with atypical chest pain was referred to Emergency Department for clinical evaluation. Vital signs at admission were stable, and the physical exam was normal.

ECG showed only biphasic T wave in V3 (*Panel A*). Transthoracic echocardiography showed hypokinesis of the mid-anterior septum, anterior, and inferior walls with preserved ejection fraction (*Panel B*). Relevant laboratory tests included elevated high sensitivity troponin-I of 3176 ng/L (normal < 38 ng/L) and B-type natriuretic peptide of 276 pg/mL (normal < 100 pg/mL).

The patient was diagnosed with non-ST segment elevation myocardial infarction and addressed to invasive coronary angiography, with the evidence of non-obstructive coronary artery disease. Left ventriculography identified hypokinesia of mid-left ventricular segments and hyperkinesia of apical and basal segments (*Panel C*).

Cardiac magnetic resonance (CMR) confirmed hypokinesia of all mid-ventricular segments and preserved contraction of apical and basal segments (*Panel D*). At tissue characterization, while traditional T2-weighted (*Panels E* and *F*) and late gadolinium enhancement (*Panel G*) imaging did not show abnormalities, mapping sequences detected a marked increase of native T1 (up to 1285 ms, *Panel H*), T2 (up to 82 ms, *Panel I*), and extracellular volume (ECV) (up to 41%, *Panel J*) values at all mid-ventricular segments, particularly at anterior, septal, and inferior walls. All these findings were related to the presence of oedema, then supporting the diagnosis of mid-ventricular Takotsubo cardiomyopathy (TC).

The apical ballooning pattern in TC is seen in the majority of patients. A very small group of TC presents with atypical features such as mid-ventricular patterns. In this case, CMR was crucial to identify not only peculiar wall motion abnormalities but also to detect, thanks to mapping sequences, the underlying tissue damage, thus leading to the correct diagnosis.

